# Gene–Diet Interactions in Diabetes Mellitus: Current Insights and the Potential of Personalized Nutrition

**DOI:** 10.3390/genes16050578

**Published:** 2025-05-14

**Authors:** Angeliki Kapellou, Effie Salata, Dimitrios Miltiadis Vrachnos, Sevastiani Papailia, Spiros Vittas

**Affiliations:** iDNA Laboratories, 7 Kavalieratou Taki, 14564 Athens, Greece; angie.kapellou@idna.gr (A.K.); effie.salata@idna.gr (E.S.); dimitris.vrachnos@idna.gr (D.M.V.); sevastiani.papailia@idna.gr (S.P.)

**Keywords:** nutrigenetics, polymorphisms, glycemia, SNP, personalized nutrition

## Abstract

Type 2 diabetes mellitus (T2DM) remaina significant global health challenge, with its increasing prevalence and associated complications contributing to high morbidity and economic burden. Genetic factors play a crucial role in T2DM susceptibility, yet individual responses to dietary interventions vary widely, emphasizing the importance of gene–diet (G × D) interactions. This review synthesizes the current literature on the genetic basis of T2DM and the role of G × D interactions in shaping individual responses to diet. We examine the genetics implication in T2DM risk and modulation by dietary factors, with a focus on the potential of Nutrigenetics in guiding personalized nutrition (PN) strategies. Moreover, the clinical implications of these interactions for the personalized prevention and management of T2DM are explored, highlighting the promise of tailoring dietary recommendations based on genetic profiles. Critical research gaps, including the need for diverse and longitudinal studies, the integration of multi-omic data, and the inclusion of digital health technologies in PN are discussed. Finally, future directions for the field are outlined, advocating for more inclusive, large-scale studies to optimize PN approaches for diverse populations and improve the efficacy of T2DM prevention and management. This review underscores the potential of an individualized, genetically informed dietary approach in modulating the global burden of T2DM.

## 1. Introduction

Diabetes mellitus is a chronic metabolic disease defined primarily by hyperglycemia. Diabetes is typically classified into categories, including autoimmune type 1 diabetes, diabetes secondary to pancreatic injury, diabetes from specific genetic disorders, and the broad category of type 2 diabetes mellitus (T2DM), which involves impaired insulin secretion and variable insulin resistance [1]. Among these, monogenic diabetes stands out for its well-defined etiology and minimal influence from environmental factors. Resulting from a single gene mutation in one of over 40 known genes, it accounts for 1–5% of diabetes cases in youth and young adults [2,3]. The main subtypes include neonatal diabetes mellitus (NDM) and maturity-onset diabetes of the young (MODY), which presents across multiple generations and follows an autosomal dominant inheritance pattern [2]. Rarer forms include multisystem syndromes, lipodystrophy and severe insulin resistance without obesity [4].

In contrast, T2DM is a multifactorial disease, driven by the complex interplay of multiple genetic loci and a range of environmental and lifestyle factors such as physical inactivity, poor dietary habits, and psychosocial stressors [5,6]. Unlike the rare monogenic forms, T2DM is far more prevalent and represents a major global health burden, contributing to significant morbidity, mortality, and economic costs due to its associated microvascular and macrovascular complications [7]. Despite widespread public health efforts, the prevalence of T2DM is steadily increasing, with projections estimating that over 850 million individuals will be affected by 2050 [1].

Over the past two decades, advances in genetics have enabled the identification of hundreds of genetic loci associated with T2DM, primarily through genome-wide association studies (GWASs). However, these genetic variants often confer only modest individual risk, underscoring the importance of environmental modifiers [8,9]. Among these, diet plays a central role not only in the prevention of T2DM but also in its progression and management [10]. Increasing evidence suggests that the effect of dietary factors on glycemic regulation and diabetes risk may be significantly influenced by underlying genetic variation—a phenomenon described as the gene–diet (G × D) interaction [11,12].

In an epidemiological context, G × D interactions refer to a scenario where the joint effect of genetic predisposition and dietary exposure on T2DM risk differs from the sum or product of their individual effects [13]. Biologically, such interactions imply that diet and genetic factors co-participate in shared pathways influencing glucose metabolism, insulin sensitivity, or inflammatory processes [12,13]. The identification of these interactions holds immense promise for the development of personalized nutrition (PN) approaches, where dietary recommendations are tailored based on individual genetic profiles to achieve optimal metabolic outcomes [14].

The role of gene–environment interactions in the etiology and progression of T2DM has been explored in various reviews, particularly in relation to lifestyle factors such as physical activity, diet, and weight management [15]. While genetic predisposition contributes significantly to T2DM risk, genetics alone are not determinative. It is the interaction between genetic variants and modifiable factors—especially diet—that plays a critical role in shaping individual disease risk and outcomes [9]. Building on this foundation, recent advances in Nutrigenetics and multi-omics have enhanced our understanding of how specific dietary components interact with genetic variants to influence T2DM risk and progression. Therefore, the aim of this review is to provide an updated and focused synthesis of the current evidence on G × D interactions in T2DM. It further explores implications for PN, highlights key methodological challenges, and outlines future directions for integrating genetic insights into personalized dietary strategies for diabetes care.

## 2. Genetic Basis of T2DM

Although the heritability of T2DM is well established, with estimates ranging from 30% to 70% [16], the underlying genetic architecture is complex and polygenic, involving numerous loci that influence insulin secretion, β-cell function, and insulin sensitivity [17]. Unlike monogenic forms of diabetes, T2DM does not result from a single genetic mutation but rather from the cumulative effect of many genetic variants. The advent of GWASs has vastly expanded the known genetic landscape of T2DM. Early GWAS efforts, such as those by the MAGIC and DIAGRAM consortia, focused primarily on European populations and led to the identification of dozens of susceptibility loci [18,19]. By 2021, over 700 independent T2DM-associated loci from large-scale multi-ancestry analyses had been discovered [20].

Despite this remarkable progress, findings from the largest T2DM GWAS meta-analysis to date—spanning five ancestral groups—show that these common variants explain only about 20% of the disease’s heritability and still do not outperform family history in predictive power [21]. Importantly, family history remains one of the strongest and most accessible predictors of T2DM, with first-degree relatives facing up to a threefold higher risk of developing the disease [22]. To enhance clinical relevance, researchers have turned to polygenic risk scores (PRSs). While early PRSs had limited predictive value [23], recent models using multi-ancestry data have shown improved risk discrimination [21]. For example, Vujkovic et al. demonstrated that individuals in the top 10% of the PRS distribution had over fivefold higher T2DM risk compared to the lowest 10%. However, the accuracy of PRSs has been reported to vary by ancestry, performing best in European populations (AUC = 0.66) and worst in African Americans (AUC = 0.57) [24].

Although current PRS models offer only modest improvements over traditional clinical predictors, they hold potential for identifying individuals at elevated risk earlier in life—potentially before conventional risk factors emerge. Still, whether this genetic insight can effectively guide preventative interventions to reduce future T2DM incidence remains to be elucidated. Based on expert consensus, the current limitations in predictive accuracy and lingering scientific uncertainties argue against the widespread use of PRSs in T2DM genetic screening [25].

## 3. Gene–Diet Interactions in T2DM

G × D interactions refer to the dynamic interplay between an individual’s genetic makeup and dietary exposures, which can influence the risk, progression, and clinical outcomes of complex diseases such as T2DM [26]. Rather than acting in isolation, genes and diet often interact in ways that modify metabolic responses to food intake. Emerging research underscores that the effectiveness of dietary interventions in T2DM can vary significantly based on an individual’s genetic makeup [27]. These G × D interactions form the cornerstone of Nutrigenetics, a field that explores how genetic variations influence nutrient responses [28]. In the context of T2DM, understanding these interactions is pivotal for developing PN strategies aimed at prevention and management.

Recent studies have highlighted the role of PRSs in modulating the impact of dietary patterns on T2DM risk. For instance, a study on three US cohorts demonstrated that individuals with a higher PRS for T2DM who adhered to healthy dietary patterns, such as the Mediterranean diet, exhibited a significantly reduced risk of developing T2DM compared to those with lower adherence [29]. This suggests that even among genetically predisposed individuals, diet quality can modulate disease risk.

The ASPIRE-DNA pilot study assessed the impact of DNA-personalized dietary advice on individuals with non-diabetic hyperglycemia over 26 weeks. Participants were randomly assigned to standard care, DNA-based dietary advice, or DNA-based advice via an app and wearable. While no significant changes in fasting plasma glucose (FPG) were seen at 6 weeks, both DNA intervention groups showed significant reductions in FPG and HbA1c at 26 weeks compared to standard care. There was also a trend toward reduced progression to T2DM [30]. These results suggest that gene-based PN may be more effective than standard care, though benefits appear over a longer period and should be interpreted cautiously.

A study from the NHLBI TOPMed program investigated G × D interactions affecting the relationship between macronutrient intake and glycemic traits in a diverse sample of 33,187 non-diabetic individuals from ten cohorts. An isocaloric substitution of carbohydrates for fat was associated with modest reductions in glycemic markers such as HbA1c. One significant G × D interaction was identified; a common variant (rs79762542, 78 kb upstream of the *FRAS1* gene), enriched in individuals of African ancestry, showed a genotype-specific association between carbohydrate intake and lower HbA1c levels [12]. The study underscores the potential for ancestry-specific G × D interactions and suggests that very large sample sizes are needed to reliably detect such interactions, especially when accounting for common measurement errors in dietary assessment.

A genome-wide interaction study (GWIS) used data from 136,880 multi-ancestry participants in the UK Biobank to investigate how genetic variation modifies the effects of adherence to a Mediterranean diet on glycemic (HbA1c) and inflammatory (hsCRP) biomarkers. While the Mediterranean diet generally reduces T2DM risk, the study found significant inter-individual variability likely influenced by G × D interactions. Gene-level analyses identified *LRRC24* and *CCDC40* as significantly interacting with Mediterranean diet adherence to affect HbA1c, particularly driven by alcohol and fish intake, respectively. Additionally, variants in *LIN9* were found to modulate the relationship between nut consumption and HbA1c, with the strongest interaction observed in individuals completing multiple dietary assessments [31]. These findings highlight gene-specific modifiers of dietary effects on glycemic health and support the role of PN based on genetic profiles.

A large prospective study from the UK Biobank (*n* = 142,271) investigated whether a low-inflammatory diet reduces the risk of T2DM and whether it can modify the effect of genetic predisposition. Using an inflammatory diet index (IDI) derived from dietary intake and CRP levels, participants were followed for up to 15 years. Both normoglycemic and prediabetic individuals with low IDI scores had significantly reduced T2DM risk compared to those with high IDI scores (HR = 0.71 and 0.81, respectively). A low-inflammatory diet delayed T2DM onset by over 2 years in normoglycemic individuals. Additionally, there was a strong interaction between genetic risk and diet: those with both low genetic risk and low-inflammatory diets had up to a 74% reduced risk of developing T2DM [32]. These findings suggest that anti-inflammatory diets can significantly reduce and delay the onset of T2DM, particularly when combined with favorable genetic profiles.

A study from the Korean Genome and Epidemiology Study Cohort analyzed the relationship between dietary patterns rich in antioxidant nutrients and T2DM, using the Recommended Food Score (RFS). The analysis also explored G × D interactions. The findings indicated that the RFS was positively associated with T2DM risk. When participants were grouped based on their diet quality (low, intermediate, and high) and their PRS for T2D, those in the high genetic risk group who also had poor dietary patterns were more likely to develop T2DM [33]. This highlights the combined effect of poor diet and high genetic risk on the development of T2DM, suggesting that individuals with high genetic susceptibility to T2DM may be particularly affected by their dietary choices.

Despite converging on the relevance of G × D interactions in T2DM, the reviewed studies differ significantly in methodology. Most are prospective cohort designs but vary in population, dietary assessment tools (e.g., food frequency questionnaires, inflammatory diet indices, or dietary scores), and the genetic data analyzed—ranging from the PRS to GWAS scans. These inconsistencies in study design, population ancestry, gene coverage, and diet quality metrics contribute to the heterogeneity in the findings and should be considered when interpreting the results and assessing the translational potential.

These findings, summarized in Table 1, support the concept of Nutrigenetics—the study of how individual genetic variation affects response to nutrients. PN, guided by genetic insights, holds promise for more effective, individualized strategies in the prevention and management of T2DM.

## 4. Benefits of G × D Interactions in T2DM

The clinical application of G × D interactions in T2DM presents a promising avenue for enhancing prevention and treatment strategies. Rather than adopting a one-size-fits-all approach, PN leverages individual genetic information to tailor dietary guidance, with the goal of aligning nutritional recommendations with individual biological makeup. Crucially, this individualized approach may also facilitate more meaningful behavior change. As T2DM is largely preventable and modifiable through behavior, the integration of genetic insights into clinical care offers an innovative, patient-centered pathway for improving metabolic health and fostering sustained self-management.

### 4.1. Identification of High-Risk Individuals

In clinical practice, identifying genetically high-risk individuals can serve as a catalyst for personalized monitoring and early dietary or lifestyle interventions [34]. This approach shifts the paradigm from reactive treatment to proactive risk reduction, aligning with public health priorities. Genetic screening enables the early identification of individuals at elevated risk of obesity and T2DM [35].

GWASs have uncovered numerous loci associated with T2DM susceptibility, such as *TCF7L2* and *FTO*, which influence pathways related to insulin secretion, adiposity, and glucose metabolism [36,37]. Notably, carriers of the *TCF7L2* rs7903146 or rs12255372 variants demonstrate significantly impaired β-cell function and an increased risk of T2DM, even in the absence of obesity [38,39].

The early identification of these genetic risk profiles allows for targeted interventions in at-risk individuals—often before the manifestation of hyperglycemia—thereby enabling primary prevention. The utility of this approach is particularly relevant in younger or lean individuals who may not be flagged by conventional risk factors, yet carry high-risk alleles that predispose them to metabolic dysfunction [40].

Moreover, genetic risk can provide significant improvements in T2DM risk stratification, especially when combined with lifestyle factors such as diet [41]. By focusing resources on those with the highest genetic risk, healthcare providers can optimize the impact of PN plans, potentially improving metabolic outcomes and reducing the incidence of T2DM [42].

### 4.2. Motivation and Adherence

The integration of genetic information into dietary counseling offers a promising opportunity to enhance the effectiveness of lifestyle interventions for T2DM. G × D interactions provide valuable insights into how individuals with specific genetic variants may respond differently to macronutrient composition, dietary patterns, and nutrient timing. While this biological tailoring is the cornerstone of PN, its clinical utility ultimately hinges on its ability to facilitate meaningful, sustained behavior change.

A key premise is that individuals are more likely to engage with lifestyle interventions when they perceive the recommendations as both personally relevant and scientifically grounded in their unique biology [43]. Although the delivery of personalized genetic risk information is often perceived as motivating, earlier research has shown that receiving such information alone rarely results in lasting improvements in health behaviors [43]. More recent findings, however, suggest that when genetic feedback is incorporated into broader, behaviorally informed PN interventions, participants exhibit greater improvements in dietary intake compared to those receiving standard dietary advice [44].

The limited behavioral impact of complex genetic risk feedback may stem from the lack of integration with psychological frameworks. Behavior change is inherently multifactorial and requires more than the provision of information. To be effective, PN strategies must align with evidence-based behavior change theories—such as the Health Belief Model, or the Self-Determination Theory—that emphasize individual autonomy, perceived competence, and readiness to act [45,46]. Without such foundation, the motivational potential of genetic information may be underutilized or even misinterpreted.

When thoughtfully designed, gene-based nutrition advice can significantly enhance patient engagement. In the Food4Me study, participants who received gene-based PN recommendations demonstrated greater improvements in dietary quality and reported higher perceived relevance of the information [47,48]. Moreover, studies indicate that individuals receiving tailored feedback often perceive the information as more trustworthy and actionable, which may increase commitment to change [49]. Psychological models such as the Self-Determination Theory support these findings by emphasizing the importance of autonomy, competence, and relatedness in health behavior change. PN strategies can satisfy these needs by providing biologically personalized, yet understandable and actionable, recommendations that help individuals feel in control of their health journey [50].

The incorporation of genetic insights can also refine not only the content but the delivery of interventions. Frameworks such as the Nutrigenomics Care Map emphasize personalized, patient-centered counseling that accounts for genetic predispositions, behavioral readiness, and motivational context [51]. This structured approach supports sustained engagement by framing genetic feedback in ways that enhance autonomy, self-efficacy, and long-term adherence.

In summary, gene-informed dietary counseling holds the potential to fine-tune nutritional advice while simultaneously acting as a psychological catalyst for behavior change. This dual advantage—biological precision and motivational resonance—positions Nutrigenetics as a compelling tool in the prevention and management of T2DM. However, to fully realize this potential, future PN interventions must move beyond risk disclosure alone and embrace behaviorally grounded, theory-driven models that empower individuals to translate genetic insights into lasting lifestyle change.

## 5. Research Gaps and Future Directions

Despite significant advancements in understanding G × D interactions in the context of T2DM, this field remains in its early stages. Several critical gaps continue to hinder the translation of Nutrigenetic findings into clinical or public health practice. Key challenges include the scarcity of long-term, prospective studies, limited ancestral diversity in research cohorts, the insufficient integration of digital health technologies, and a need for robust methodologies that address the complexity of dietary behavior, gene expression, and metabolic outcomes. Addressing these gaps is essential to fully realize the potential of PN in preventing and managing T2DM.

### 5.1. Study Designs

To date, most studies investigating the role of Nutrigenetics in T2DM risk have been either cross-sectional or based on short-term dietary interventions. While these approaches offer valuable snapshots of G × D interactions, they fall short in establishing causality and in capturing the dynamic, long-term interplay between genetic predisposition, dietary habits, and glycemic outcomes [52]. This limitation impedes the ability to formulate precise, time-sensitive dietary recommendations that align with an individual’s genetic and metabolic profile.

Robust longitudinal cohort studies are crucial to bridge this gap. Such studies enable researchers to observe how G × D interactions evolve over years or decades, offering insight into the cumulative effects of dietary exposures in genetically diverse populations. Longitudinal data can reveal critical windows of susceptibility or opportunity across the life course—from early development to adulthood—where dietary interventions might have the greatest preventive impact [53,54].

Some prospective cohorts, such as the Nurses’ Health Study and the Health Professionals Follow-up Study, have begun to integrate genomic data alongside dietary assessments and long-term metabolic outcomes [38]. These efforts are shedding light on how genetics modify individual responses to dietary patterns, such as the Mediterranean diet in relation to T2DM onset [55].

Furthermore, initiatives such as the UK Biobank and the EPIC (European Prospective Investigation into Cancer and Nutrition) cohort provide an unparalleled opportunity to integrate large-scale genetic data with detailed longitudinal lifestyle and dietary data. These platforms are particularly well-positioned to support genetic risk modeling and multi-omics analyses that include genomics, epigenetics, metabolomics, and gut microbiome composition [56,57].

However, challenges persist. Longitudinal studies require significant time, financial investment, and participant retention strategies. Additionally, the complexity of dietary assessment methods and the inter-individual variability in diet reporting and metabolism pose further analytical hurdles [58]. Advances in wearable tracking, digital dietary logging, and biomarker-based validation methods may help address some of these issues, allowing for more accurate and scalable data collection over time [59].

Ultimately, longitudinal studies are essential to move beyond correlation and into the realm of prediction and prevention in PN and T2DM. By capturing the temporal dimension of G × D interactions, these studies hold the key to designing truly personalized dietary interventions that can adapt to the genetic, metabolic, and lifestyle shifts across the human lifespan.

### 5.2. Population Diversity

A persistent and significant limitation in genetics research is the overwhelming underrepresentation of non-European populations. Over 80% of GWASs to date have been conducted in individuals of European descent, despite these individuals comprising less than 20% of the global population [60,61]. This disparity severely restricts the generalizability of current findings, limiting their clinical utility in non-European populations and raising critical concerns about equity and effectiveness in PN.

The implications of this lack of diversity are profound. Genetic variants influencing dietary response and metabolic disease risk often differ in frequency, expression, or effect across ancestries [62]. For example, alleles associated with insulin resistance or lipid metabolism may be more prevalent or functionally distinct in African, South Asian, or Indigenous populations compared to European cohorts. Applying European-derived risk scores or dietary recommendations across all populations risks reinforcing existing health disparities rather than reducing them [63].

Incorporating diverse populations into Nutrigenetic research is essential to uncover ancestry-specific gene–diet interactions. For instance, recent studies in African American and Hispanic/Latino populations have identified unique loci associated with glucose metabolism and T2DM risk that are absent or weakly associated in European datasets [64,65]. Similarly, population-specific gene–nutrient interactions—such as differential responses to fiber, saturated fat, or dietary sodium—underscore the importance of tailoring nutritional recommendations in a culturally and biologically informed way [66].

Efforts to improve representation are underway. Global consortia such as the All of Us Research Program (USA), H3Africa, China Kadoorie Biobank, and Latin American Genomic Consortium aim to create more ethnically inclusive datasets to power multi-ancestry genomic discovery [67,68]. These initiatives are particularly relevant to T2DM, a condition with significant prevalence and variation across racial and ethnic groups, often shaped by interactions between genetics, environment, socioeconomic factors, and dietary traditions. Enhancing population diversity in research is not only a scientific imperative but also an ethical obligation. Without deliberate inclusivity, PN risks becoming another layer of systemic inequality—offering benefits predominantly to those already advantaged in health systems and research infrastructure [69]. Community engagement, equitable data sharing, and culturally sensitive research designs must become central to the future of nutrigenomics.

The genetic landscape of diverse populations is further shaped by dietary habits, highlighting how G × D interactions may influence health outcomes. A well-characterized example is the copy number variation (CNV) of the *AMY1* gene, encoding salivary amylase. Populations with historically high-starch diets, such as agricultural societies (e.g., the Japanese), exhibit increased *AMY1* copy numbers, enhancing starch digestion. In contrast, groups with traditionally low-starch intake—such as the Yakut of Siberia—show fewer copies [70,71]. Similarly, lactase persistence (LP), the continued expression of the lactase enzyme into adulthood, exemplifies dietary adaptation to dairying [72,73]. While LP is prevalent in Northern Europeans, it remains rare in East Asian, Indigenous Australian, and Native American populations. Although primarily attributed to SNPs near the *LCT* gene, recent evidence implicates epigenetic regulation, including DNA methylation and miRNA activity, in modulating lactase expression [74,75,76]. These findings underscore the need for genotype- and culture-aware approaches in PN.

In summary, expanding Nutrigenetics research to encompass diverse populations in terms of genetics and ethnic dietary habits is crucial to both the discovery of novel ancestry-specific interactions and the development of equitable, effective PN strategies for T2DM prevention and management.

### 5.3. Multi-Omic Integration

The complexity of T2DM necessitates a comprehensive approach to understanding its multifactorial nature. The latest evidence suggests that integrating various omic layers—genomics, epigenomics, transcriptomics, proteomics, and metabolomics—may offer a more holistic perspective on disease pathogenesis and potential therapeutic targets.

Among these, epigenomics examines reversible, often heritable DNA and chromatin modifications that regulate gene expression. It plays a key role in mediating G × D interactions, linking nutritional inputs to metabolic outcomes via mechanisms such as DNA methylation, histone modification, and non-coding RNA regulation [77]. Dietary factors can remodel the epigenome, influencing genes involved in insulin signaling, glucose metabolism, and inflammation. For instance, high-fat diets have been associated with the aberrant methylation of genes regulating glucose homeostasis, contributing to insulin resistance and β-cell dysfunction [77,78]. Moreover, early-life and maternal nutrition can program long-term metabolic trajectories through epigenetic reconfiguration, with transgenerational effects [79]. Therefore, epigenetic biomarkers are being explored as predictors of T2DM risk, supporting the utility of epigenomic profiling in preventive strategies.

A recent editorial by Frontiers in Endocrinology underscored the importance of integrated multi-omic studies in understanding metabolic disorders such as T2DM. The editorial highlighted how combining different omic layers can elucidate disease mechanisms, identify biomarkers, and inform therapeutic strategies [80]. A recent study utilized a multi-omic strategy to construct a gene regulatory network associated with T2DM. By integrating data from genomic, transcriptomic, and epigenomic categories, the researchers identified ten key regulatory genes and pathways implicated in the disease, providing insights into its molecular underpinnings. The genes included *PSMB9*, *COL1A1*, *COL4A1*, *HLA-DQB1*, *COL3A1*, *IRF7*, *COL5A1*, *CD74*, *HLA-DQA1*, and *HLA-DRB1*, with several validated through ROC analysis, proteomics, and qPCR [81].

A more recent study applied machine learning to integrate multi-omics data from human pancreatic islets—including RNA sequencing, DNA methylation, SNPs, and phenotype data—from 110 donors (~30% with T2DM). In their analysis, the researchers achieved high predictive performance for T2DM (91 ± 15% accuracy; AUC = 0.96 ± 0.08). The approach identified novel biomarkers across omic layers, such as *SACS* and *TXNIP* methylation, *OPRD1* and *RHOT1* expression, and a SNP near *ANO1*. These findings offer new insights into the molecular interplay underlying β-cell dysfunction in T2D and highlight the promise of multi-omics machine learning in disease prediction and biomarker discovery [82].

Another study assessed whether combining genomic, proteomic, metabolomic, and clinical biomarkers could improve T2DM prediction beyond established clinical models. Using data from a nested case-cohort (N = 1105), the authors found that proteomic markers alone offered the strongest predictive power among single-omic layers (C-index = 0.82). However, the most notable improvement came from integrating the top features across all omic layers, raising the model’s predictive performance to a C-index of 0.87. In individuals with normal HbA1c (<42 mmol/mol), where prediction is most valuable, the PRS drove the greatest gain (Δ C-index = 0.06), yet even high-PRS individuals had relatively low absolute risk over 20 years. Overall, while multi-omic data marginally improved T2D risk prediction, the findings underscore the limitations in using the PRS for widespread preventive screening [83].

Collectively, these studies highlight the transformative potential of integrated multi-omics to unravel the complex biological networks underlying T2DM. By bridging diverse molecular layers—genomic, epigenomic, transcriptomic, proteomic, and metabolomic—this systems-level approach offers unprecedented insights into disease mechanisms. While direct multi-omic investigations of G × D interactions in T2DM remain scarce, the frameworks established by these studies lay the essential groundwork. Integrating dietary exposure data with multi-omics could illuminate how specific nutrients influence gene expression, metabolic pathways, and microbiome composition in genetically susceptible individuals. This would mark a critical step toward deciphering the biological basis of Nutrigenetic interactions and advancing PN strategies for T2DM prevention and management.

### 5.4. Digital Tools and PN for T2DM

The integration of digital health technologies into the management of T2DM is rapidly advancing. Tools such as mobile applications, wearables, and Artificial Intelligence (AI)-driven systems have the potential to enhance the delivery, monitoring, and efficacy of PN interventions by enabling real-time data collection, personalized feedback, and scalable implementation.

A meta-analysis investigated the use of mobile health applications (mHealth apps) for managing T2DM in adults, focusing on current usage patterns, future interest, and patient attitudes. The results showed that 35% of individuals currently use mHealth apps for disease self-management, while 57% expressed interest in using these apps in the future. However, 39% of participants lack confidence in the effectiveness of these applications [84]. A related study examined the factors influencing mHealth adoption among patients with T2DM. The study found that the perceived ease of use and perceived usefulness significantly shaped user attitudes, while digital health literacy and a positive orientation toward technology further enhanced acceptance [85].

A 2023 review explored how AI is revolutionizing healthcare, particularly in the fields of nutrition and clinical biochemistry. AI-enabled tools for tracking health metrics, such as glucose levels, body weight, and calorie intake, are becoming essential for managing T2DM. Moreover, these technologies show potential for integrating genetic data, paving the way for more tailored dietary interventions based on individual genomic profiles and metabolic characteristics [86]. Another review emphasized the pivotal role of wearable technologies, including continuous glucose monitors (CGMs) and smartwatches, in advancing glucose monitoring and diabetes care. It highlighted the integration of AI and multi-omics data—such as genomics, proteomics, and microbiome analyses—to personalize care and deliver predictive analytics. These technologies facilitate the tracking of key factors such as physical activity, dietary intake, medication adherence, and glucose levels. Future developments may include smart glasses and next-generation AI models to further enhance data integration and real-time decision-making [26].

Despite the promise of these technologies, several challenges have been identified. Issues such as data accuracy, algorithmic bias, privacy concerns, and the need for validation across diverse populations remain critical. Additionally, disparities in digital literacy and technology access risk widening existing health inequities if not thoughtfully addressed [87,88]. One persistent limitation is the “law of attrition,” whereby digital health tools often face higher dropout rates than traditional clinical interventions, with user engagement declining over time. While low-burden tools, such as wearables and photo-based dietary apps, may increase convenience, they can also reduce opportunities for users to develop meaningful awareness of their dietary habits. Studies have shown mixed results regarding their effectiveness compared to higher-burden approaches such as manual food tracking [88].

Nevertheless, when carefully designed and implemented, digital tools and PN strategies offer substantial promise for managing metabolic diseases such as T2DM. Long-term success will depend on sustained user engagement, robust behavior modification frameworks, and equitable access to digital health solutions.

### 5.5. Framework for Clinical Integration

Despite growing interest in PN and gene-informed dietary interventions, the translation of Nutrigenetic insights into clinical care for T2DM remains limited. To facilitate this transition, structured, evidence-based frameworks are needed to guide healthcare professionals in the ethical and practical application of genetic information.

One such model is the Nutrigenomics Care Map, an evidence-informed decision-support tool designed to assist clinicians in integrating genetic data within a patient-centered counseling approach [51]. This framework emphasizes the integration of multi-omics data, lifestyle factors, and behavioral readiness, enabling risk stratification and the development of tailored nutrition goals that reflect both genetic susceptibility and individual motivation. It also encourages shared decision-making and the use of personalized feedback to support autonomy and self-efficacy—principles shown to be vital for PN effectiveness [89].

The clinical integration of PN further requires investment in infrastructure, including access to validated genetic testing, secure data management systems, and digitally enabled decision-making tools [89]. Critically, the role of the practitioner is central: registered dietitians and healthcare professionals must be trained in genomic literacy to accurately interpret results and translate them into actionable, comprehensible advice. Interdisciplinary collaboration—among dietitians, genetic counselors, behavioral scientists, and clinicians—is essential to ensure interventions are both effective and contextually appropriate [90].

Ethical considerations are foundational to these frameworks. The use of genetic data must uphold patient autonomy, privacy, and equity while avoiding deterministic messaging or overpromising outcomes. Clear communication about the implications and limitations of genetic findings is vital to building trust and avoiding misinterpretation, particularly in populations with varying levels of health literacy [91].

As PN begins to move toward clinical mainstreaming, the field of implementation science will be essential in evaluating which delivery models work best in real-world settings, how training can be standardized, and how outcomes—both behavioral and metabolic—can be meaningfully tracked [89]. While scientific readiness is advancing, system-level preparedness remains a key determinant of success.

In summary, integrating Nutrigenetics into clinical care is not simply a scientific challenge but an operational and ethical one. Models such as the Nutrigenomics Care Map offer a starting point, but broader institutional support, interdisciplinary education, and patient-centered design will be essential to realize the full promise of gene-informed diabetes care.

**Figure 1:**
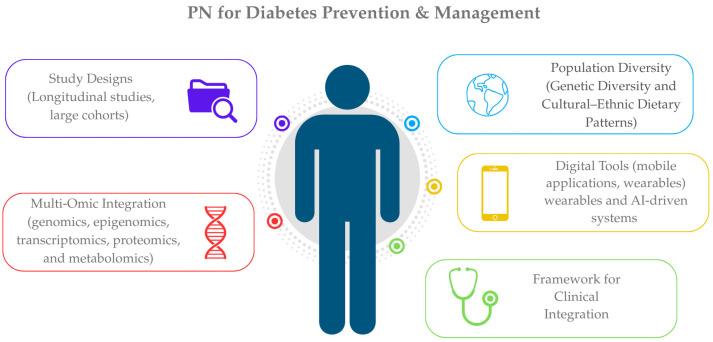
Key components informing precision nutrition strategies for T2DM prevention and management.

Looking forward, future research must prioritize large-scale, multi-ethnic, and longitudinal studies that leverage advanced omic and digital technologies. Such efforts are essential not only for validating G × D interactions but also for translating them into actionable and scalable public health strategies aimed at T2DM prevention and management through PN (Figure 1).

## 6. Conclusions

The interplay between genetic variation and diet is a critical determinant of diabetes risk and management. Advances in genomics and nutrition science have paved the way for personalized dietary recommendations that may optimize glycemic control and prevent disease onset. While challenges in the implementation and evidence translation remain, the integration of G × D interactions into clinical practice holds transformative potential for the future of diabetes care.

## Figures and Tables

**Table 1:** Summary of key gene–diet interaction studies in T2DM risk and management.

Study/Authors (Year)	Population	Sample Size (n)	Study Design	Genes/SNPs	Dietary Factors	Key Findings
Apio et al., 2023 [33]	Korean	10,038	Cohort	GWAS ^1^ scan	Food scores based on 46 food items	Food scores were positively associated with T2D risk (OR = 1.11, 95% CI: 1.03–1.20, *p* = 0.009 and OR = 1.10, 95% CI: 1.02–1.19, *p* = 0.011). Interaction analyses between food scores and SNPs identified 12 candidate genes (e.g., *CACNA2D3*, *RELN*, *DOCK2*) and implicated the adipocytokine signaling pathway as the most strongly associated with T2D, involving 32 key genes including *STAT3*, *IRS1*, *AKT1–3*, and *ADIPOR2*. Individuals at high genetic risk were more susceptible to the adverse effects of lower diet quality.
Karvela et al., 2024 [30]	UK	148	RCT ^1^	12 SNPs	Standard care vs. DNA-based diet based on participants’ three highest risk results	DNA-based dietary intervention significantly reduced fasting plasma glucose (−0.019 mmol/L, *p* = 0.01) and HbA1c (−0.038, *p* = 0.04) compared to standard care at 26 weeks but not earlier.
Merino et al., 2022 [29]	US	35,759	Cohort	PRS based on 67 SNPs	AHEI and DASH	Each 1 SD increase in the PRS was associated with a 29% higher T2DM risk (RR = 1.29, 95% CI: 1.25–1.32, *p* < 0.001), and each 10-unit decrease in the AHEI score conferred a 13% higher risk (RR = 1.13, 95% CI: 1.09–1.17, *p* < 0.001). Poor diet quality increased T2DM risk by ~30% regardless of genetic risk (*P* interaction = 0.69), with no significant interaction between diet and genetics (*P* interaction = 0.30).
Westerman, Meigs and Manning, 2023 [31]	UK	136,880	Cohort	GWAS scan	MDS	G × D interactions influencing HbA1c were primarily driven by alcohol and fish intake, with a significant interaction between nut intake and *LIN9* variants (*p* = 8.8 × 10^−8^). The top SNP (rs9729447) showed that the inverse association between nut intake and HbA1c was attenuated in major allele carriers.
Westerman et al., 2023 [12]	African American, American Indian, Asian, White, and Hispanic/Latino	33,178	Cohort	GWAS scan	Isocaloric substitution of carbohydrates for fats	A higher carbohydrate intake was modestly associated with lower glycemic traits (−0.013% HbA1c per 250 kcal substitution). A significant G × D interaction was identified for *rs79762542*, enriched in African ancestry, where the inverse carbohydrate–HbA1c association was observed only in major allele homozygotes and replicated in the UK Biobank.
Yang et al., 2023 [32]	UK	142,271	Cohort	PRS based on 424 SNPs	IDI ^1^ score based on the sum of 34 food items (16 anti-inflammatory and 18 pro-inflammatory)	Over a median 8.4-year follow-up, low IDI scores were associated with reduced T2DM risk in both normoglycemic (HR = 0.71) and prediabetic (HR = 0.81) individuals. A low-inflammatory diet delayed T2DM onset by ~2.2 years in normoglycemia and ~1.1 years in prediabetes. Joint analysis showed up to a 74% reduced risk among those with both low genetic risk and low IDI scores.

^1^ AHEI: Alternate Healthy Eating Index; DASH: Dietary Approaches to Stop Hypertension; G × D interactions: gene–diet interactions; GWAS: genome-wide association study; IDI: inflammatory diet index; MDS: Mediterranean diet score; PRS: polygenic risk score; RCT: randomized controlled trial; SNPs: single nucleotide polymorphisms.
